# Blueberries Improve Pain, Gait Performance, and Inflammation in Individuals with Symptomatic Knee Osteoarthritis

**DOI:** 10.3390/nu11020290

**Published:** 2019-01-29

**Authors:** Chen Du, Amy Smith, Marco Avalos, Sanique South, Keith Crabtree, Wanyi Wang, Young-Hoo Kwon, Parakat Vijayagopal, Shanil Juma

**Affiliations:** 1 Department of Nutrition and Food Science, Texas Woman’s University, Denton, TX 76204, USA; cdu@twu.edu (C.D.); asmith60@twu.edu (A.S.); ssouth@twu.edu (S.S.); kcrabtree@twu.edu (K.C.); pvijayagopal@twu.edu (P.V.); 2 School of Health Promotion and Kinesiology, Texas Woman’s University, Denton, TX 76204, USA; mavalos1@twu.edu (M.A.); YKwon@twu.edu (Y.-H.K.); 3 Center for Research Design and Analysis, Texas Woman’s University, Houston, TX 77030, USA; WWang@twu.edu

**Keywords:** blueberry, knee osteoarthritis, pain, polyphenols, gait, inflammation

## Abstract

Osteoarthritis (OA) is the most common joint disorder in the world and is the most frequent cause of walking related disability among older adults in the US, which brings a significant economic burden and reduces quality of life. The initiation and development of OA typically involves degeneration or progressive loss of the structure and function of articular cartilage. Inflammation is one of the major drives of the progression of OA. Dietary polyphenols have been studied for their anti-inflammatory properties and potential anabolic effects on the cartilage cells. Blueberries are widely consumed and are high in dietary polyphenols, therefore regular consumption of blueberries may help improve OA. The purpose of the present study was to examine the effect of freeze dried whole blueberries on pain, gait performance, and inflammation in individuals with symptomatic knee OA. In a randomized, double-blind trial, adults age 45 to 79 with symptomatic knee OA, were randomized to either consume 40 g freeze-dried blueberry powder (*n* = 33) or placebo powder (*n* = 30) daily for four months. Blood draws and assessment of pain and gait were conducted at baseline, two months, and four months. Western Ontario McMaster Osteoarthritis Index (WOMAC) questionnaires were used to assess pain and GAITRite^®^ electronic walkway was used to evaluate gait spatiotemporal parameters. WOMAC total score and sub-groups, including pain, stiffness, and difficulty to perform daily activities decreased significantly in the blueberry treatment group (*p* < 0.05), but improvement of WOMAC total score and difficulty to perform daily activities were not observed in the placebo group. Normal walking pace single support percentage for both limbs increased (*p* = or < 0.007), while double support percentage for both limbs decreased in the blueberry treatment group (*p* = or < 0.003). No significant changes were observed in plasma concentrations of tumor necrosis factor (TNF)-α, interleukin (IL)-1β, IL-6, IL-10, IL-13, matrix metalloproteinases (MMP)-3, MMP-13, and monocyte chemoattractant protein-1 (MCP-1) in both treatment groups. However, an increasing trend for IL-13 concentration and a decreasing trend in MCP-1 concentration were noted in the blueberry group. The findings of this study suggest that daily incorporation of whole blueberries may reduce pain, stiffness, and difficulty to perform daily activities, while improving gait performance, and would therefore improve quality of life in individuals with symptomatic knee OA.

## 1. Introduction

Osteoarthritis (OA) can be defined by symptoms such as pain and decreased flexibility. It is typically evaluated or classified through radiographic X-ray [1]. It often affects joint cartilage and/or underlying bones, involving the articular surfaces of synovial joints [2]. Symptomatic OA is defined as the presence of radiographic OA in combination with symptoms attributable to OA [1]. The primary symptoms of OA are joint pain, aching, and stiffness. OA is the most common joint disorder in the world and is the most frequent cause for walking-related disability among older adults in the United States [1]. It has been identified as an inflammatory disease of synovial joints and due to the symptoms and pathogenesis related structural changes of OA, it impacts one’s gait performance, therefore limiting physical activities [3]. One of the latest OA prevalence studies conducted by Barbour et al. reported over 54 million adults (22.7% of the US population) suffering from doctor diagnosed OA and it is projected to affect 78.4 million adults by 2040 [4]. Factors that contribute to the development of knee OA include systemic risk factors and local biomechanical risk factors. The systemic risk factors include age, gender, race/ethnicity, genetics, obesity, osteoporosis, bone density, and nutrition, while the local biomechanical risk factors include joint injury, certain occupations, physical activities, limb-length inequality, neuromuscular factors, bone characteristics, and joint space [5]. Among all risk factors, obesity, aging and female gender appear to be the most significant ones [6].

Current treatments for OA include pharmacologic, non-pharmacologic, and surgical intervention. Common pharmacological agents used include acetaminophen and other anti-inflammatory medications [7,8]. Long-term use of these pharmacological agents has potential adverse effects that can lead to serious consequences, including gastrointestinal bleeding and adverse cardiac effects [9,10]. Current pharmacological interventions only have a palliative benefit relieving the symptoms of OA while not treating the underlying problem of cartilage breakdown. Nutraceutical supplements, such as glucosamine and chondroitin, have been used to treat OA and reduce symptoms. However, there is currently no convincing information for the efficacy of such supplements in treating OA. Therefore, alternative, natural, and effective methods of reducing symptoms along with promoting healing and repair of cartilage tissue are warranted.

There is a growing body of research demonstrating a relationship between increased consumption of dietary polyphenols and their protective benefit in reducing risk for chronic human diseases, such as cardiovascular disease, hypertension, cancers, diabetes, certain infectious diseases, and osteoarthritis [11]. The proposed mechanism by which polyphenols reduce the risk of chronic human disease involves their ability to accept electrons from free radicals, thereby disrupting chain oxidation reactions, and increasing cellular antioxidative capacity [12]. Numerous in vitro studies and animal studies, and a small number of clinical trials have demonstrated polyphenols’ chondroprotective and anti-inflammatory actions [13,14,15,16,17,18,19,20]. Inflammation plays a major role the initiation and the development of OA. Studies suggest that inflammation occurs at the earliest stage of OA and contributes to the progression of OA [21,22]. Inflammatory cytokines and MMPs such as TNF-α, IL-1β, IL-6, MMP-3, and MMP-13 have been shown to be elevated in patients with OA [23,24,25] and contribute to the progression of OA [26,27]. On the other hand, anti-inflammatory cytokines such as IL-10 and IL-13 have been shown to have chondroprotective effects which slow down the progression of OA [28,29].

Blueberries are consumed worldwide. Besides their pleasing taste, they are a significant source of polyphenols [30]. The major polyphenols found in blueberries are anthocyanins. Both in vitro and in vivo studies on anthocyanin effects in joint tissue have shown an anti-inflammatory effect [31]. Studies have shown that flavonoids increase the cartilage anabolic activity by enhancing certain factors such as insulin-like growth factor-1 (IGF-1), osteocalcin, bone morphogenetic protein (BMP), etc. [32,33]. A few clinical trials have also shown that blueberries have a potential anti-inflammatory effect [34,35] and could improve functionality measured by gait parameters in older adults [36]. However, there have been no studies with blueberries investigating their effects on pain reduction, functionality improvement, and inflammation in individuals with OA. This study investigated the effects of daily consumption of whole blueberries in individuals with symptomatic OA.

## 2. Materials and Methods

### 2.1. Study Design

Using a double-blind randomized placebo-controlled pre-test and post-test design, a total of 63 men and women, between the ages of 45 and 79, with self-reported symptomatic osteoarthritis were recruited through the local Denton community, Texas Woman’s University, and local orthopedic clinics. Recruited participants agreed to not take any cyclooxygenase-2 (COX-2) inhibitors, chondroitin sulfate, glucosamine sulfate, or glucosamine hydrochloride powder, all of which are known to have anti-inflammatory effects and/or influence the symptoms of knee pain. In addition, participants agreed not to consume any blueberry products or blueberries during the study.

### 2.2. Recruiting, Inclusion/Exclusion Criteria

Identification of potential subjects included a short screening questionnaire completed by phone. This screening questionnaire included questions on demographic information, smoking history, medical history, current medications/supplements, special diet, food allergies, and blueberry consumption. The inclusion criteria were men and women aged 45 to 79 experiencing knee pain, and in relatively healthy condition. The exclusion criteria were men and women who smoke more than one pack of cigarettes per day, have uncontrolled diabetes, who were on an insulin regimen that does not allow additional carbohydrate as part of a routine diet, who have congestive heart failure, who have knee replacements on both knees, or those who were using prescribed COX-2 inhibitors, chondroitin sulfate, glucosamine sulfate, or glucosamine hydrochloride powder and were not willing to stop taking these medications/supplements during the study period. Those who were allergic to blueberries were also excluded from participation.

### 2.3. Intervention

Eligible men and women were randomly assigned to one of two groups, a treatment group (*n* = 33) or a placebo group (*n* = 30). The treatment group received 40 g of freeze-dried whole blueberry powder daily, provided by the US Highbush Blueberry Council (Folsom, CA, USA). The powder was constituted by merely extracting water, while maintaining the composition of whole blueberries, then milling to fine powder [37]. The powder was packaged in 20 g packets, with participants in the treatment group requested to consume two packets per day. The placebo group was also asked to consume 40 g of control powder daily, divided into 20 g packages, consumed twice a day. The placebo powder was constituted of maltodextrins to mimic the carbohydrate composition of whole blueberries, but without whole blueberries. The appearance of the placebo powder, along with energy content, are similar to blueberry powder. Participants in the treatment and control groups were instructed to reconstitute their respective powders in 10–12 ounces of water, immediately followed by consumption. Participants were on the respective regimen for a period of four months. The study protocols were approved by the Institutional Review Board at Texas Woman’s University.

Qualified subjects were invited to the study site for three visits during the four-month study period, which included a baseline visit, midpoint visit (at two months), and final visit (at four months). At the baseline visit, qualified subjects were provided with a written consent form and informed on all aspects of the study. After consent forms were signed, research proceeded according to procedure. The procedures in each visit included anthropometric measurements including weight, height, leg length, and blood pressure, along with obtaining fasting blood samples, performing gait test, and filling out a WOMAC questionnaire.

Treatment compliance was tracked using a calendar for daily consumption of blueberry or placebo powder. Calendars were provided to participants at baseline, midpoint, and final visits, and participants were instructed to bring the calendar back on the following visits. Participants were also called for follow up to ensure compliance and to address any concerns.

### 2.4. Pain and Gait Assessment

Pain was assessed by using the WOMAC questionnaire at baseline, midpoint, and final visits. These questionnaires have been validated and have been used in many research projects studying symptomatic knee osteoarthritis [38]. Gait and balance were analyzed using a GAITRite^®^ system (CIR Systems, Inc., Franklin, NJ, USA), a portable electronic walkway. The 10-meter GAITRite^®^ system was set up and utilized by trained research personnel. Subjects were instructed to walk three trials on the walkway at self-elected (usual) speed and instructed to walk another three trials on the walkway at the fastest speed that they can walk without running. Twenty seconds of rest/pause were allowed between each trial. An average of the three trials for each gait velocity was recorded for analysis. The gait parameters measured included cadence, velocity, right and left step and stride length, right and left single and double support percentage to one gait cycle. The GAITRite^®^ system has been validated and used as a reliable tool to assess a myriad of spatio-temporal gait parameters [39].

### 2.5. Biochemical Variables

Overnight fasting venous blood was collected in ethylenediaminetetraacetic acid (EDTA) at baseline, midpoint, and final visits by a trained phlebotomist. Blood was centrifuged at 3000 g for 10 min to separate plasma, which then was aliquoted into collection tubes and stored at −80 °C for subsequent analysis of IL-1β, IL-6, TNF- α, IL-10, IL-13, MMP-3, MMP-13, and MCP-1. Magnetic bead multiplex ELISA kits from Millipore were used to analyze blood biomarkers of inflammation. Three different panels were conducted. A human high sensitivity T cell panel was used to analyze IL-1β, IL-6, TNF- α, IL-10, and IL-13 with inter-assay CVs (coefficient of variation) of <20%, a human MMP panel was used for analyzing MMP-3 and MMP-13 with inter-assay CVs of <20%, and a human metabolic hormone panel was used for analyzing MCP-1 with inter-assay CVs of <25%. Luminex 200 software (Luminex Corporation, Austin, TX, USA) was used to analyze the raw data.

### 2.6. Statistical Analysis

Descriptive statistics were calculated for all variables, comprising means, standard deviations, minima, and maxima for all continuous variables. Frequencies and percentages were calculated for all categorical variables. Distributions of the response variables were examined to determine if statistical tests of hypotheses based on the assumption of normality were being met, and that parametric testing is appropriate. Extreme outliers were investigated for technical or clerical error. Baseline differences of dependent variables were tested using independent sample *t*-tests. Although dependent variables in each area are related to each other, due to the small sample size, repeated measures (ANOVAs) were conducted to examine outcome differences of pain, stiffness, difficulties to perform daily activities, gait performance parameters, and inflammation biomarkers between treatments over time at baseline, midpoint, and final. Most variables are normally distributed, and some variables contain outliers. Therefore, the analyses were done on outlier-removed data as well. The data was analyzed using SPSS 25.0.0. (IBM, Armonk, NY, USA) All *p*-value ≤ 0.05 were considered statistically significant.

## 3. Results

### 3.1. Demographics

A total of 103 individuals were screened for participation in the study. Of those screened, 63 qualified individuals were scheduled for the baseline visit. Over the course of the study, there were 14 individuals who withdrew from the study due to issues such as taste and palatability of treatment, lack of interest, lack of compliance, or conflicts in study visit scheduling. A total of 49 participants completed the study. Demographic data associated with study participants is provided in Table 1. The majority of the participants in this study were female, who comprised 71% of the whole population at final point.

**Figure 1:**
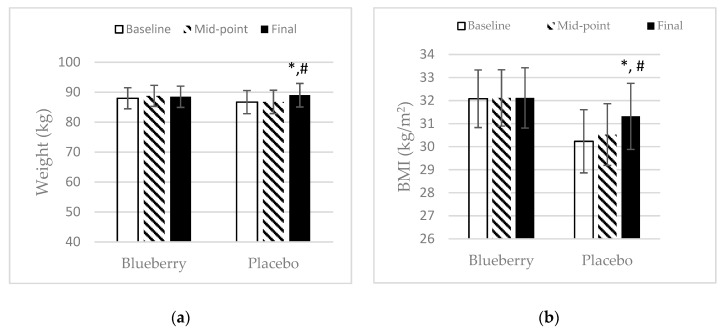
Effect of blueberry and placebo on weight and BMI: (**a**) Weight. Mean ± SEM. N = 27 for the blueberry group, *N* = 22 for the placebo group. *, significance as compared to baseline (*p* < 0.05); #, significance as compared to midpoint (*p* < 0.05). The mean difference between final point and baseline is a net change of +2.31 kg. (**b**) Mean ± SEM. *N* = 24 for the blueberry group, *N* = 20 for the placebo group. Outliers were omitted. *, significance as compared to baseline (*p* < 0.05); #, significance as compared to midpoint (*p* < 0.05). The mean difference between final point and baseline is a net change of +1.10 kg/m^2^.

**Figure 2:**
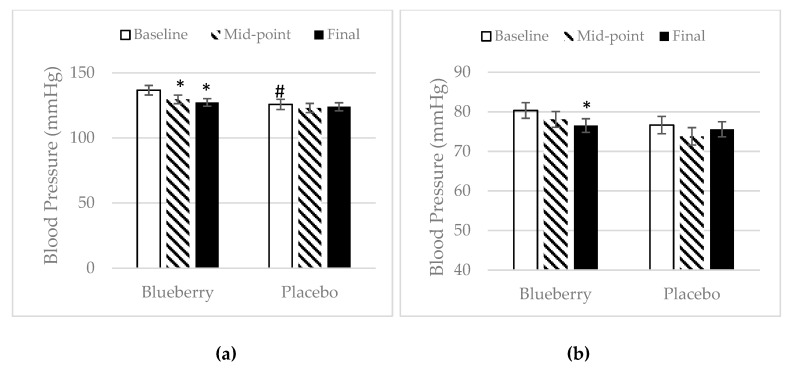
Effect of blueberry and placebo on systolic and diastolic blood pressure. (**a**) Systolic blood pressure. Mean ± SEM. *N* = 25 for the blueberry group, *N* = 22 for the placebo group. Outliers were omitted. *, significance as compared to baseline (*p* < 0.05); #, Placebo Baseline vs. Blueberry group is significantly different (*p* < 0.05). (**b**) Diastolic blood pressure. Mean ± SEM. *N* = 27 for the blueberry group, *N* = 22 for the placebo group. Outliers were omitted. *, significance as compared to baseline (*p* < 0.05).

For body weight, height, and BMI, there was no significant difference between treatment groups at baseline. The body weight increased significantly at final point over baseline and midpoint in the placebo group, whereas the blueberry group maintained their body weight throughout the study, as shown in (Figure 1a). In the blueberry group, participants maintained their BMI throughout the study period, while participants in the placebo group had a significant increase in BMI at final point over baseline and midpoint (Figure 1b). A significant difference in systolic blood pressure between the two treatment groups was noted at baseline, but not of diastolic blood pressure. In the blueberry group, participants’ systolic blood pressure decreased significantly at midpoint over baseline and remained at the reduced level at final point (Figure 2a). Also, participants’ diastolic blood pressure dropped significantly at final point over baseline in the blueberry group (Figure 2b). There were no significant changes of systolic and diastolic blood pressure in the placebo group throughout the study. Besides the baseline differences in systolic blood pressure, there was no significant difference between the blueberry and the placebo group in regard to weight, height, BMI, or systolic and diastolic blood pressure, at any time point.

### 3.2. WOMAC

**Figure 3:**
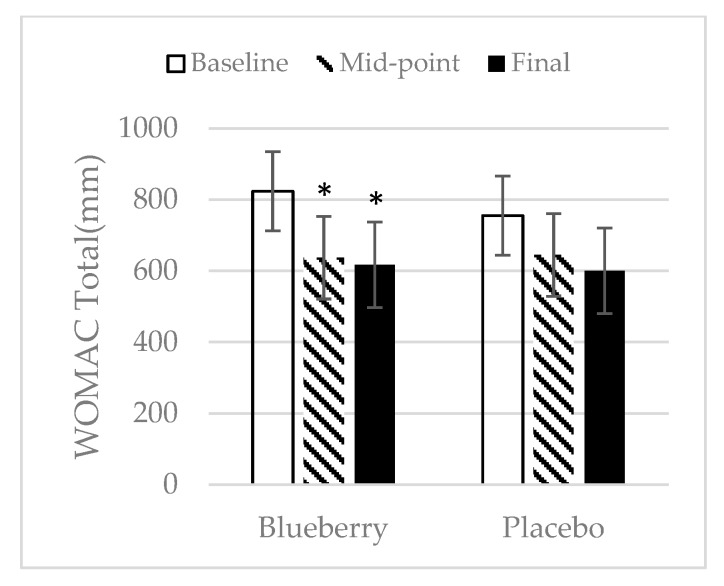
Western Ontario McMaster Osteoarthritis Index (WOMAC) total. Mean ± SEM. *N* = 22 for the blueberry group, *N* = 22 for the placebo group. *, significance as compared to baseline (*p* < 0.05).

**Figure 4:**
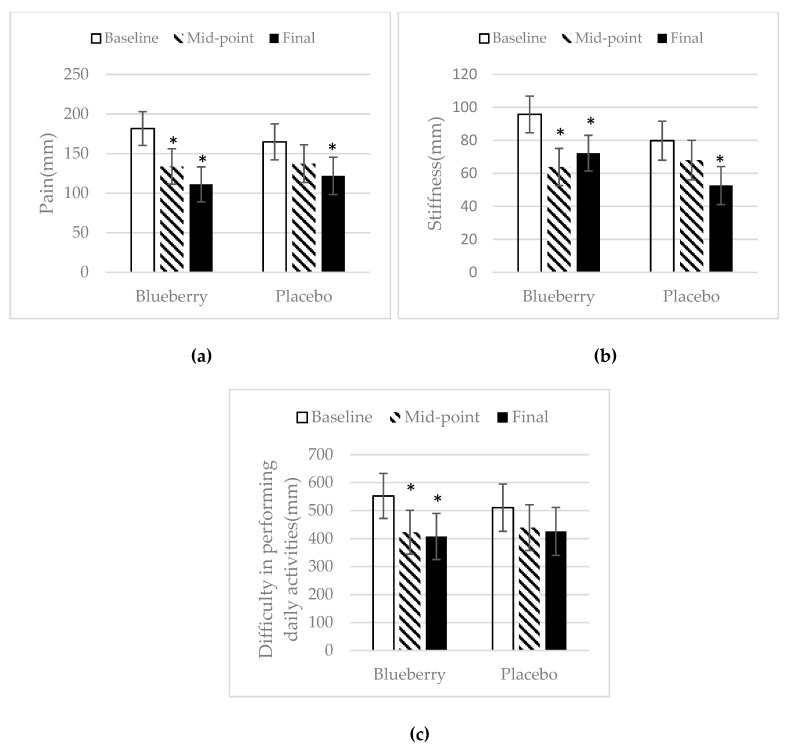
WOMAC sub-scores: (**a**) WOMAC sub-group pain. Mean ± SEM. *N* = 25 for the blueberry group, *N* = 22 for the placebo group. *, significance as compared to baseline (*p* < 0.05). (**b**) WOMAC sub-group stiffness. Mean ± SEM. *N* = 25 for the blueberry group, *N* = 22 for the placebo group. *, significance as compared to baseline (*p* < 0.05). (**c**) WOMAC sub-group difficulty to perform daily activity. Mean ± SEM. *N* = 24 for the blueberry group, *N* = 22 for the placebo group. *, significance as compared to baseline (*p* < 0.05).

The WOMAC questionnaire was used to assess pain, stiffness, and difficulty to perform daily activities during the study. There was no difference in baseline WOMAC total score, pain, stiffness, or difficulty to perform daily activities between the two treatment groups. The total WOMAC score significantly decreased at midpoint over baseline and at final point over baseline for the blueberry group (Figure 3). There was no change in the total WOMAC score in the placebo group throughout the study. In the blueberry group, pain decreased significantly at midpoint over baseline and continued to decrease at final point over baseline. In the placebo group, pain was significantly reduced at final point as compared to baseline (Figure 4a). For stiffness, in the blueberry group, there was a significant decrease at midpoint over baseline and at final point over baseline. In the placebo group, there were no significant changes in stiffness from baseline to midpoint, but a significant decrease in stiffness at final point over baseline was noted (Figure 4b). Significant decreases in difficulty to perform daily activities were observed in the blueberry group for midpoint and final point over baseline. There was no significant change of difficulty in performing daily activities in the placebo group throughout the study (Figure 4c). There was no difference between the blueberry and placebo group in pain, stiffness, and difficulty to perform daily activities at any time point of the study. Female only data was also analyzed for WOMAC and the results were consistent with the data analyzed for both groups.

### 3.3. Gait Performance

In the blueberry group, normal paced velocity increased significantly at final point over baseline, and final point over midpoint (*p* < 0.05). There was a significant increase in normal paced walking left single support percentage to one gait cycle at midpoint over baseline (*p* < 0.05), and at final point over both baseline and midpoint (*p* < 0.05). At the same time, the decrease in the normal paced walking left limb led double support percentage to one gait cycle was noted at midpoint over baseline (*p* < 0.05) and final point over baseline (*p* < 0.05). A similar pattern was also noted in the normal paced right limb led single and double support percentage to one gait cycle in the blueberry group. Meanwhile, in the placebo group, there was a significant increase in the normal paced walking left single support percentage at midpoint (*p* < 0.05) and final point (*p* < 0.05) over baseline, but there was no change in the normal paced right single support percentage. For double support for both limbs in the placebo group, there was no change at final point over baseline. There were no changes in fast-paced single and double support percentage to one gait cycle for either limb in both groups at final point over baseline. A baseline difference was noted on the normal paced walking double support percentage to one gait cycle only in the right limb between the two groups. Measurements of gait parameters are shown in Table 2. In addition, data analysis of gait parameters was conducted for female only data, no additional findings were observed in the female population.

### 3.4. Inflammation

**Figure 5:**
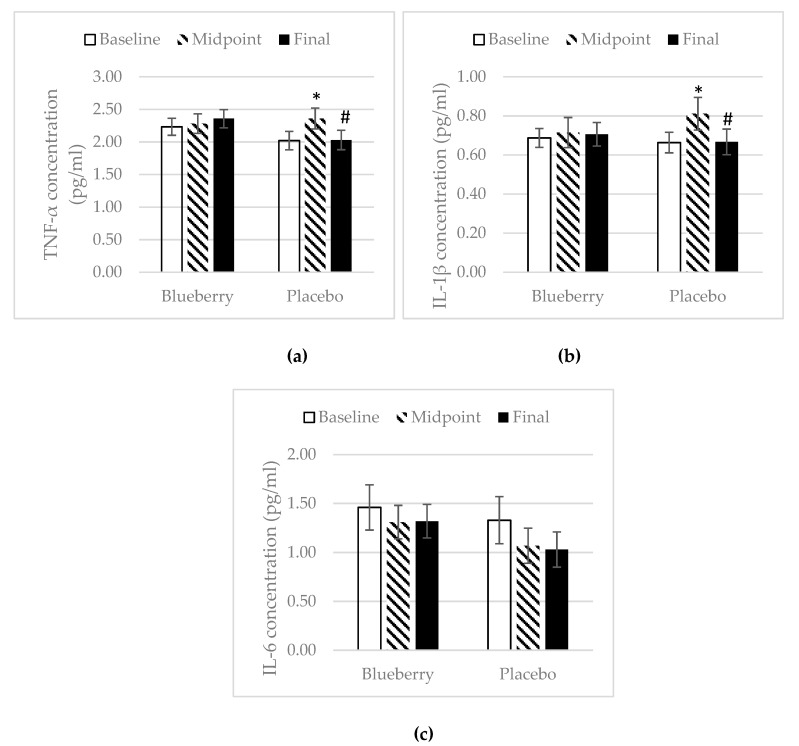
Tumor necrosis factor (TNF)-α, interleukin (IL)-1β, and IL-6. Mean ± SEM. (**a**) TNF-α, *N* = 26 for the blueberry group, *N* = 21 for the placebo group; (**b**) IL-1β, *N* = 26 for the blueberry group, *N* = 22 for the placebo group; (**c**) IL-6, *N* = 22 for the blueberry group, *N* = 20 for the placebo group. *, significance as compared to baseline (*p* < 0.05); #, significance as compared to midpoint (*p* < 0.05).

**Figure 6:**
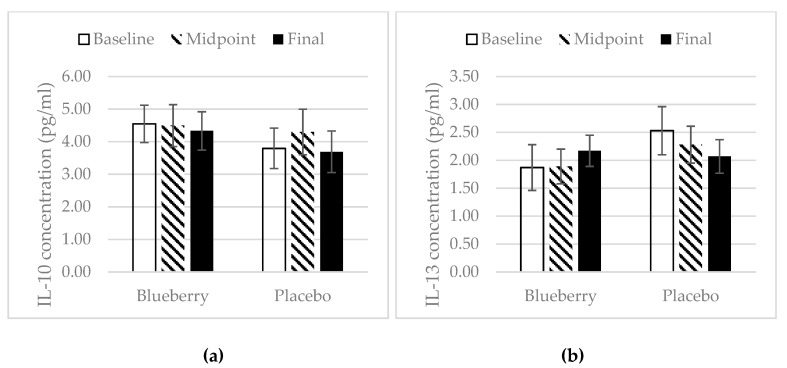
IL-10 (**a**) and IL-13 (**b**). Mean ± SEM. *N* = 26 for the blueberry group, *N* = 22 for the placebo group. No significant changes over time and between groups at any time point. However, an increase of 0.30 pg/mL in plasma concentration of IL-13 was noted at final point as compared to baseline in the blueberry group, while a decrease of 0.46 pg/mL in concentration was noted at final point as compared to baseline in the placebo group.

**Figure 7:**
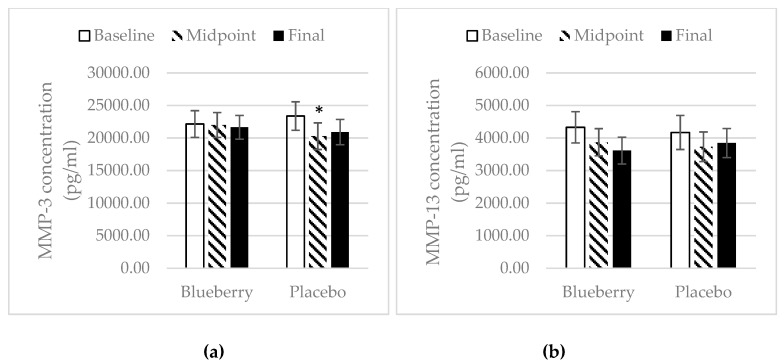
Matrix metalloproteinases (MMP)-3. Mean ± SEM. (**a**) MMP-3, *N* = 26 for the blueberry group, *N* = 22 for the placebo group. *, significance as compared to baseline (*p* < 0.05); (**b**) MMP-13. Mean ± SEM. *N* = 26 for the blueberry group, *N* = 22 for the placebo group. No significant changes over time and between groups at any time point.

**Figure 8:**
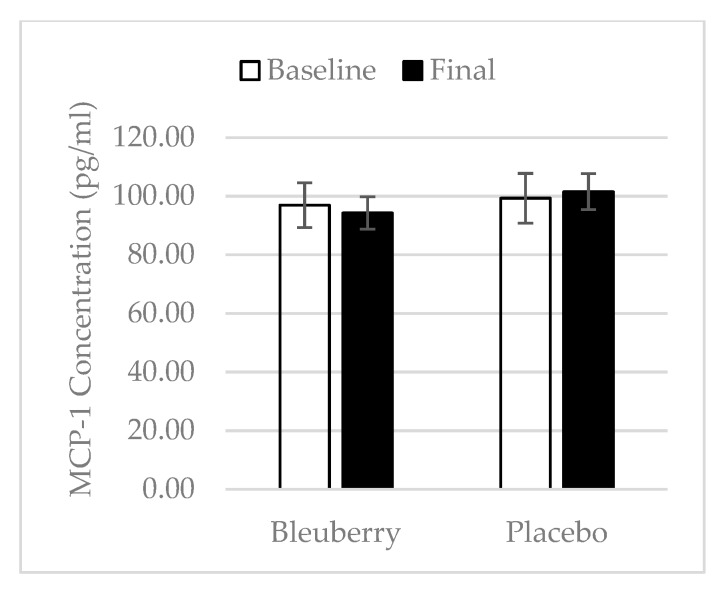
Monocyte chemoattractant protein-1( MCP-1). Mean ± SEM. *N* = 27 for the blueberry group, *N* = 22 for the placebo group. No significant changes over time and between groups at any time point. Plasma concentration of MCP-1 decreased by 2.65 pg/mL in the blueberry group at final point as compared to baseline, while in the placebo group, the concentration increased by 2.23 pg/mL.

There were no changes in plasma concentration of inflammatory biomarkers in the blueberry group over the study period. In the placebo group, there was a significant increase in the plasma concentration of TNF-α at midpoint over baseline, but there was a decrease at the final point over midpoint (Figure 5a). There was also an increase in IL-1β at midpoint over baseline with a decrease at final point over midpoint, and no change overall for final point over baseline (Figure 5b). IL-6 concentration remained consistent over the study periods in the placebo group (Figure 5c). For the anti-inflammatory biomarkers, the plasma concentration of IL-10 and IL-13 stayed the same over the study period for both treatment groups. However, there was an overall increase in concentration of IL-13 at final point over baseline (+0.30 pg/mL) in the blueberry group, while there was an overall decrease in the placebo group at final point over baseline (−0.46 pg/mL) (Figure 6b). The concentration of MMP-3 and MMP-13 in the blueberry group showed a decrease at midpoint over baseline and continued to decrease at final point, but the decreases at both midpoint and final point over baseline did not reach statistical significance. The plasma concentration of MMP-13 stayed the same over the study period in the placebo group (Figure 7b). The MMP-3 concentration decreased at midpoint over baseline but rebounded at the final point (Figure 7a). A decrease in concentration of MCP-1 was observed in the blueberry group, while an increase in MCP-1 was observed in the placebo group (Figure 8). However, the changes did not reach significance. No significant changes of the inflammatory biomarkers, anti-inflammatory biomarkers, and MMPs were observed in the female only population.

## 4. Discussion

The findings of the present study demonstrated that freeze-dried whole blueberry powder consumption for a period of four months results in a reduction in pain, stiffness, and difficulty to perform daily activities, an improved normal walking paced gait performance, and a favorable impact to certain inflammatory and anti-inflammatory biomarkers. A growing body of research has demonstrated a positive relationship between increased consumption of polyphenols and its protective effects in reducing chronic human disease risk [11]. The positive effects are attributed to its antioxidant and anti-inflammatory properties [40]. Both in vitro and in vivo studies have investigated polyphenols’ protective effects on osteoarthritis. Polyphenols from green tea extract, turmeric, red wine, citrus fruits, and pomegranate have been extensively investigated with many studies showing some level of anti-inflammatory and cartilage protective polyphenol effects [41,42,43,44,45].

Results from the WOMAC identified decreased pain and stiffness, and improved ability to perform daily activities in the blueberry group after consuming freeze-dried whole blueberry after 60 days of treatment, with the effects continuing to 120 days. These results are encouraging as pain and stiffness are the major symptoms for individuals suffering from OA, limiting their functional ability and thus lowering quality of life. However, improvements during the blueberry treatment were not significantly different from the placebo group at any time point. In a randomized double-blind crossover study investigating the efficacy of a tart cherry juice blend in treatment of knee OA, investigators observed a similar significant reduction in WOMAC scores in the tart cherry juice treatment group. Once again, the differences between treatment and placebo were not significant [46].

There was significant improvement in normal paced walking gait performance in the blueberry group, indicated by increased cadence, velocity, step and stride length for both limbs, increased single support percentage to one gait cycle and decreased double support percentage to one gait cycle for both limbs. The improvement happened as early as 60 days and continued to improve through the end of the treatment period. Stride length and cadence are correlated with classification of knee OA severity. Lower stride length and cadence correlated with higher grade OA as classified by the Kellgren and Lawrence evaluation system [47]. Single limb support is strongly correlated with WOMAC pain and function, as well as velocity and step length, which correlates with WOMAC pain and function [48]. Due to double limb support and single limb support collectively forming one gait cycle, when single limb support percentage to one gait cycle decreases, double limb support percentage to one gait cycle increases. The findings of gait performance in normal walking pace are consistent with the WOMAC score changes. Similarly, no significant differences were observed between the blueberry group and placebo group at any time point throughout the study. These results may be compared with other plant-based nutraceuticals which have been studied for their functions of improving gait. Javaheri et al. found that treating mice with sulforaphane—commonly found in cruciferous vegetables—for three months, led to greater symmetry in gait [49]. Innes et al. investigated feeding extracts of Indian and Javanese turmeric, *Curcuma domestica*, and *Curcuma xanthorrhiza* to dogs with OA for eight weeks, and examined gait improvement post treatment. No significant improvement in gait performance was observed in the treatment group [50]. However, Schrager et al. found similar results in older adults (age > 60 years old) as compared to the current study. In particular, Schrager et al. found that consumption of two cups of frozen blueberries a day for six weeks improved gait speed, reduced the number of step errors during single task adaptive gait, and increased gait speed during dual task adaptive gait [36].

In contrast to the placebo group, the inflammatory cytokines TNF-α, IL-1β, and IL-6 did not change in the blueberry group throughout the study, but a non-significant decrease in concentration of MCP-1 was noted at final point as compared to baseline. TNF-α, IL-1β, and IL-6 have been found to increase during the worsening of OA and contribute to the progression of OA. MCP-1 has been shown to contribute to the initiation and progression of OA [51]. Thus, blueberry treatment was able to prevent worsening of inflammation. The anti-inflammatory effects of polyphenols have been studied extensively. Most positive results are reported in in vitro and animal studies. Shakibaei et al. showed that pretreatment of human primary articular chondrocytes with resveratrol for 4 h, followed by treatment with IL-1β and continued treatment of resveratrol results in a reduction of IL-1β induced apoptosis [52]. A limited number of randomized controlled clinical trials investigating polyphenols’ anti-inflammatory effects have reported inconsistent results [53]. Plasma concentrations of IL-10 and IL-13 as well as MMP-3 and MMP-13 did not change significantly during the study period, in either treatment group. IL-10 and IL-13 are anti-inflammatory cytokines. Very few studies have investigated the effect of polyphenols on these anti-inflammatory markers in humans. Most studies have focused on in vitro and animal studies. One in vitro study found that epicatechin gallate, epigallocatechin and epigallocatechin gallate, the major tea polyphenols, decreased the production of IL-1β and enhanced the production of IL-10 in human leukocytes [54]. In an in vitro study, Ahmed et al. showed that pomegranate extracts inhibited IL-1b-induced expression of MMP-1, MMP-3, and MMP-13 in human osteoarthritis chondrocytes [16]. In a mouse post-traumatic OA model, Leong et al. found that articular cartilage in the EGCG-treated mice exhibited reduced levels of MMP-1, -3, -8, -13, ADAMTS5, IL-1β, and TNF-α mRNA [41]. One parallel-designed, placebo-controlled clinical trial reported that intervention with an anthocyanin extract from blueberries (300 mg/d for three weeks) significantly reduced the plasma concentration of IL-4, IL-13, IL-8, and IFN-a in a group of 120 healthy men and women aged 40–74 years [55]. In one clinical trial, six-week consumption of pomegranate juice significantly decreased serum levels of MMP-13 [45]. However, no significant changes in IL-10, IL-13, MMP-3, and MMP-13 were observed in our present study, which is inconsistent with most results from in vitro and animal studies and the limited number of human trials. Factors other than polyphenols that can impact plasma concentrations of these biomarkers include dietary pattern, physical activity, or other inflammation issues. This could potentially explain why no changes were observed in the present study.

In the blueberry group, weight and BMI stayed the same throughout the study, while, in the placebo group, weight, and BMI increased significantly at final point over midpoint and baseline. One plausible explanation to this is that dietary polyphenols may affect neuroregulatory factors that control satiety, therefore mediating food intake and energy regulation [56]. In an animal study, investigators found that rats fed with blueberry extracts for six days had reduced food intake and weight reduction compared to rat fed with control meals due to satiety effects [57]. Systolic and diastolic blood pressure significantly reduced in the blueberry group at the final point over baseline, while there were no significant changes in the placebo group. The average systolic blood pressure of individuals in the blueberry group was significantly higher than in the placebo group. Participants were randomly assigned into two treatment groups, with the difference in systolic blood pressure at baseline differing by chance. Our results are consistent with other studies on the effect of polyphenols on blood pressure. Johnson et al. found that consuming 22 g of freeze-dried blueberry powder for eight weeks significantly lowered systolic and diastolic blood pressure among postmenopausal women with pre- and stage 1 hypertension [58]. Studies have also shown that compared with other types of foods, the intake of flavonoid-rich foods and drinks significantly reduces blood pressure [59,60]. In a double blind, placebo controlled parallel trial, Naruszewicz et al. reported that consumption of chokeberry flavonoid extract for a period of six weeks significantly reduced systolic and diastolic blood pressure by an average of 11 and 7.2 mmHg, respectively [61].

Concerning potential limitations to this study, the dropout rate was high, making it harder to detect significant changes. Larger sample sizes may be needed to meet the required statistical power for the study. Alternatively, intent-to-treat analysis may be used to preserve the sample size, but the analysis needs to be used with caution. In addition, most of the study participants were females, which was less representative of the whole population. This study was based on participant compliance with consumption of the blueberry or placebo powder. Calendars were provided to participants to help improve compliance, but the compliance was self-reported. Follow up calls were made in between study visits to encourage compliance. Participants were asked to mix the blueberry powder or placebo powder with water and consume right after mixing. A few participants reported that the palatability of the powder was less tolerable over time, so they mixed the powder with other drinks such milk or added the powder to their breakfast cereal, which could impact the bioavailability and absorption of polyphenols. Diet variations can potentially have an impact on the study outcomes. During the study period, participants were asked to not consume blueberry but did not have other requirements for their diet.

## 5. Conclusions

In conclusion, the findings of the present study suggest that blueberries may have positive effects on pain management and improving gait performance, and contributing to better physical functionality for OA patients. Future study designs should include a larger sample size, better treatment compliance protocols, and better dietary control. An alternative to a crossover study design may also be used. Also, more targeted inflammation biomarkers may be selected to better capture inflammation changes caused by OA progression.

## Figures and Tables

**Table 1:** Demographics of study participants

		Baseline	Midpoint	Final
Blueberry	Male (*n*)	9	9	9
	Female (*n*)	24	19	18
	Total (*n*)	33	28	27
	Avg Age	57.7 ± 1.8	57.5 ± 1.9	56.4 ± 1.9
	Avg BMI	32.1 ± 1.3	32.1 ± 1.2	32.1 ± 1.3
Placebo	Male (*n*)	7	5	5
	Female (*n*)	23	18	17
	Total (*n*)	30	23	22
	Avg Age	55.3 ± 1.5	54.5 ± 1.7	54.6 ± 1.8
	Avg BMI	30.2 ± 1.4	30.5 ± 1.3	31.3 ± 1.4
Total	Male (*n*)	16	14	14
	Female (*n*)	47	37	35
	Total (*n*)	63	51	49
	Avg Age	56.5 ± 1.2	56.2 ± 1.3	55.6
	Avg BMI	31.9 ± 0.8	31.6 ± 0.9	32.2 ± 0.9
Drop Rate (%)		NA	19%	22%

Values are in means ± SEM for age (years) and BMI (kg/m^2^). NA means not applicable.

**Table 2:** Gait parameters at baseline and following blueberry and placebo interventions for 8 weeks and 16 weeks in study participants (*n* = 27 for the blueberry group; *n* = 22 for the placebo group)

	Blueberry			Placebo		
Gait Parameters	Baseline	Midpoint	Final	Baseline	Midpoint	Final
Normal cadence (steps/min)	103.7 ± 2.4	111.3 ± 2.1 *	114.2 ± 2.46 *	108.4 ± 2.7	113.9 ± 2.3 *	117.2 ± 2.7 *
Fast cadence (steps/min)	144.0 ± 4.7	146.4 ± 3.8	146.0 ± 2.5	152.1 ± 5.2	149.3 ± 4.2	156.0 ± 4.7 ^#^
Normal velocity (cm/s)	96.3 ± 4.12	108.1 ± 3.8 *	115.3 ± 4.4 *^,#^	105.3 ± 4.6	114.9 ± 4.1 *	119.0 ± 4.9 *
Fast velocity (cm/s)	163.8 ± 8.2	165.2 ± 6.7	163.4 ± 7.0	176.7 ± 9.1	170.7 ± 7.4	179.8 ± 7.7 ^#^
Left step length normal (cm)	55.1 ± 1.6	58.0 ± 1.5 *	60.0 ± 1.5 *^,#^	58.1 ± 1.8	60.2 ± 1.6 *	60.8 ± 1.7 *
Left step length fast (cm)	67.5 ± 2.0	67.2 ± 1.9	66.9 ± 1.8	69.1 ± 2.3	68.3 ± 2.2	68.7 ± 2.0
Right step length normal (cm)	54.9 ± 1.6	58.1 ± 1.5 *	60.4 ± 1.6 *^,#^	58.1 ± 1.8	60.8 ± 1.7 *	60.7 ± 1.8 *
Right step length fast (cm)	67.2 ± 2.01	67.3 ± 1.8	66.5 ± 1.9	70.0 ± 2.3	69.3 ± 2.0	69.6 ± 2.1
Left stride length normal (cm)	110.2 ± 3.1	116.3 ± 2.9 *	120.7 ± 3.0 *^,#^	116.4 ± 3.5	121.0 ± 3.2 *	121.7 ± 3.4 *
Left stride length fast (cm)	134.9 ± 4.0	134.7 ± 3.6	133.6 ± 3.6	139.5 ± 4.4	137.4 ± 4.0	138.3 ± 4.0
Right stride length normal (cm)	110.2 ± 3.2	116.4 ± 2.9 *	120.6 ± 3.1 *^,#^	116.3 ± 3.5	121.1 ± 3.2 *	121.6 ± 3.4 *^,#^
Right stride length fast (cm)	134.8 ± 4.0	134.8 ± 3.7	133.7 ± 3.7	139.5 ± 4.5	137.8 ± 4.1	138.9 ± 4.1
Left single support normal (%)	35.3 ± 0.6	36.3 ± 0.5 *	37.0 ± 0.6 *^,#^	36.8 ± 0.6	37.6 ± 0.5 *	37.6 ± 0.5 *
Left single support fast (%)	40.4 ± 0.6	40.1 ± 0.6	40.1 ± 0.6	40.8 ± 0.7	40.3 ± 0.6	41.1 ± 0.6 ^#^
Right single support normal (%)	35.3 ± 0.5	36.6 ± 0.4 *	37.1 ± 0.4 *	36.9 ± 0.6	37.4 ± 0.4	37.6 ± 0.5
Right single support fast (%)	40.1 ± 0.6	40.4 ± 0.6	40.1 ± 0.58	41.0 ± 0.7	40.7 ± 0.6	40.8 ± 0.6
Left double support normal (%)	29.5 ± 1.0	27.2 ± 0.8 *	26.1 ± 0.8 *^,#^	26.6 ± 1.1	25.2 ± 0.9 *	25.3 ± 0.9
Left double support fast (%)	20.0 ± 1.2	19.3 ± 1.1	19.7 ± 1.1	18.1 ± 1.3	19.1 ± 1.2	17.7 ± 1.3 ^#^
Right double support normal (%)	29.5 ± 1.0	27.3 ± 0.8 *	26.1 ± 0.8 *^,#^	26.5 ± 1.1	25.3 ± 0.9 *	25.3 ± 0.9
Right double support fast (%)	19.8 ± 1.2	19.4 ± 1.1	19.8 ± 1.1	18.3 ± 1.33	19.2 ± 1.2	18.9 ± 1.2 ^#^

Values are means ± SEMs (Standard error of means) obtained from a linear mixed-effects model with time as within-subject factor and intervention group as a between-subject factor; *, significance as compared to baseline (*p* < 0.05); ^#^, significance as compared to midpoint (*p* < 0.05).
